# Consensus classification of posterior cortical atrophy

**DOI:** 10.1016/j.jalz.2017.01.014

**Published:** 2017-03-02

**Authors:** Sebastian J. Crutch, Jonathan M. Schott, Gil D. Rabinovici, Melissa Murray, Julie S. Snowden, Wiesje M. van der Flier, Bradford C. Dickerson, Rik Vandenberghe, Samrah Ahmed, Thomas H. Bak, Bradley F. Boeve, Christopher Butler, Stefano F. Cappa, Mathieu Ceccaldi, Leonardo Cruz de Souza, Bruno Dubois, Olivier Felician, Douglas Galasko, Jonathan Graff-Radford, Neill R. Graff-Radford, Patrick R. Hof, Pierre Krolak-Salmon, Manja Lehmann, Eloi Magnin, Mario F. Mendez, Peter J. Nestor, Chiadi U. Onyike, Victoria S. Pelak, Yolande Pijnenburg, Silvia Primativo, Martin N. Rossor, Natalie S. Ryan, Philip Scheltens, Timothy J. Shakespeare, Aida Suárez González, David F. Tang-Wai, Keir X. X. Yong, Maria Carrillo, Nick C. Fox, on behalf of the Alzheimer’s Association ISTAARTAtypical Alzheimer’s Disease and Associated Syndromes Professional Interest Area

**Affiliations:** a Dementia Research Centre, UCL Institute of Neurology, London, UK; b Department of Neurology, Memory & Aging Center, University of California, San Francisco, San Francisco, CA, USA; c Department of Neuroscience, Mayo Clinic, Jacksonville, FL, USA; d Cerebral Function Unit, Greater Manchester Neuroscience Centre, Salford Royal NHS Foundation Trust, Salford, UK; e Institute of Brain, Behaviour and Mental Health, University of Manchester, Manchester, UK; f Department of Neurology, VU University Medical Centre, Amsterdam Neuroscience, Amsterdam, The Netherlands; g Alzheimer Center, VU University Medical Centre, Amsterdam Neuroscience, Amsterdam, The Netherlands; h Department of Neurology, Massachusetts General Hospital and Harvard Medical School, Boston, MA, USA; i Laboratory for Cognitive Neurology, Department of Neurosciences, KU Leuven, Leuven, Belgium; j Nuffield Department of Clinical Neurosciences, University of Oxford, Oxford, UK; k Human Cognitive Neuroscience, School of Philosophy, Psychology and Language Sciences, University of Edinburgh, Edinburgh, UK; l Department of Neurology, Mayo Clinic, Rochester, MN, USA; m Center for Cognitive Neuroscience, Vita-Salute San Raffaele University, Milan, Italy; n INSERM U 1106, Institut des Neurosciences des Systèmes, Aix Marseille Université, Marseilles, France; o Departamento de Clínica Médica, Faculdade de Medicina, Universidade Federal de Minas Gerais, Belo Horizonte, Brazil; p Institute for Memory and Alzheimer’s Disease, UMR-S975, Salpêtrière Hospital, Pierre & Marie Curie University, Paris, France; q Aix-Marseille Université, INSERM, Institut de Neurosciences des Systèmes, Marseille, France; r AP-HM Hôpitaux de la Timone, Service de Neurologie et Neuropsychologie, Marseille, France; s Department of Neurosciences, University of California, San Diego, San Diego, USA; t Department of Neurology, Mayo Clinic, Jacksonville, FL, USA; u Fishberg Department of Neuroscience, Icahn School of Medicine at Mount Sinai, New York, USA; v Friedman Brain Institute, Icahn School of Medicine at Mount Sinai, New York, USA; w Clinical and Research Memory Center of Lyon, Hospices Civils de Lyon, INSERM U1028, CNRS UMR5292, University of Lyon, Lyon, France; x Department of Neurology, Regional Memory Centre (CMRR), CHU Besançon, Besançon, France; y Department of Neurology, David Geffen School of Medicine, University of California, Los Angeles, CA, USA; z Cognitive Neurology and Neurodegeneration Group, German Center for Neurodegenerative Diseases (DZNE), Magdeburg, Germany; aa Department of Psychiatry and Behavioral Sciences, Johns Hopkins University School of Medicine, Baltimore, MD, USA; bb Department of Neurology, University of Colorado School of Medicine, Aurora, CO, USA; cc Department of Ophthalmology, University of Colorado School of Medicine, Aurora, CO, USA; dd Memory Disorders Unit, Neurology Department, University Hospital Virgen del Rocio, Seville, Spain; ee Division of Neurology, University Health Network Memory Clinic, University of Toronto, Toronto, Ontario, Canada; ff Medical and Scientific Relations, Alzheimer’s Association, Chicago, IL, USA

**Keywords:** Posterior cortical atrophy, Alzheimer’s disease, Clinico-radiological syndrome, Pathophysiology, Biomarker

## Abstract

**Introduction:**

A classification framework for posterior cortical atrophy (PCA) is proposed to improve the uniformity of definition of the syndrome in a variety of research settings.

**Methods:**

Consensus statements about PCA were developed through a detailed literature review, the formation of an international multidisciplinary working party which convened on four occasions, and a Web-based quantitative survey regarding symptom frequency and the conceptualization of PCA.

**Results:**

A three-level classification framework for PCA is described comprising both syndrome- and disease-level descriptions. Classification level 1 (PCA) defines the core clinical, cognitive, and neuroimaging features and exclusion criteria of the clinico-radiological syndrome. Classification level 2 (PCA-pure, PCA-plus) establishes whether, in addition to the core PCA syndrome, the core features of any other neurodegenerative syndromes are present. Classification level 3 (PCA attributable to AD [PCA-AD], Lewy body disease [PCA-LBD], corticobasal degeneration [PCA-CBD], prion disease [PCA-prion]) provides a more formal determination of the underlying cause of the PCA syndrome, based on available pathophysiological biomarker evidence. The issue of additional syndrome-level descriptors is discussed in relation to the challenges of defining stages of syndrome severity and characterizing phenotypic heterogeneity within the PCA spectrum.

**Discussion:**

There was strong agreement regarding the definition of the core clinico-radiological syndrome, meaning that the current consensus statement should be regarded as a refinement, development, and extension of previous single-center PCA criteria rather than any wholesale alteration or redescription of the syndrome. The framework and terminology may facilitate the interpretation of research data across studies, be applicable across a broad range of research scenarios (e.g., behavioral interventions, pharmacological trials), and provide a foundation for future collaborative work.

## 1. Introduction

**Fig. 1:**
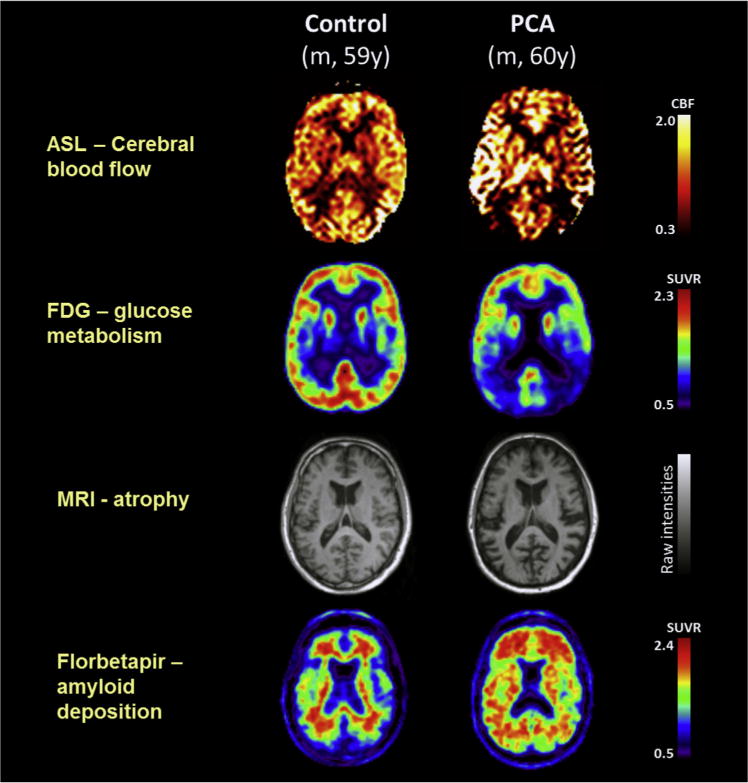
Single-participant axial images for one control participant and one patient with PCA showing cerebral blood flow (ASL), glucose metabolism (FDG-PET), atrophy (structural magnetic resonance imaging), and amyloid deposition (florbetapir-PET). For clinical purposes, ^18^F-florbetapir images should be read on a gray scale. Abbreviations: ASL, arterial spin labeling; CBF, cerebral blood flow; FDG-PET, ^18^F-labeled fluorodeoxyglucose positron emission tomography; PCA, posterior cortical atrophy; SUVR, standard uptake value ratio. For tau deposition data see Ossenkoppele et al [2]. Adapted from Lehmann et al., 2016, Figure 1.

The term posterior cortical atrophy (PCA) was coined by D. Frank Benson and colleagues to describe a series of patients with early visual dysfunction in the setting of neurodegeneration of posterior cortical regions [1] (Fig. 1). The PCA syndrome aligned with several other reports of patients with similar progressive loss of higher visual function (e.g., [3–13]). PCA typically presents in the mid-50s or early 60s with a variety of unusual visuoperceptual symptoms, such as diminished ability to interpret, locate, or reach for objects under visual guidance; deficits in numeracy, literacy, and praxis may also be apparent. Although episodic memory and insight are initially relatively preserved, progression of PCA ultimately leads to a more diffuse pattern of cognitive dysfunction.

Several single-center groups of researchers have proposed diagnostic criteria for the syndrome [14,15] or detailed inclusion criteria for individual studies (e.g., [16–18]). PCA has also been recognized and described in consensus criteria for typical and atypical Alzheimer’s disease [19,20]. These existing criteria have reasonable consistency and have proved useful in many clinical and research contexts.

However, the extant detailed descriptions of PCA are based on clinical experience at single centers and have not been deliberated or validated more widely. Present-day PCA criteria were also formulated before the development of Alzheimer’s disease (AD) pathophysiological biomarkers, and although recent AD criteria include PCA, the clinical phenotype is not described in detail and such criteria naturally do not encompass individuals with the PCA syndrome who are negative for AD pathophysiological biomarkers. Some inconsistencies exist among the core features described, with the Tang-Wai but not Mendez criteria excluding individuals with early Parkinsonism or hallucinations, while Mendez but not Tang-Wai stipulates the relative preservation of verbal fluency [14,15]. Such inconsistencies are mirrored explicitly or implicitly in the application of terminology, with the term PCA sometimes being used as a descriptive clinical (syndrome level) term and sometimes as a diagnostic (disease level) label. For example, some researchers consider PCA primarily or solely as an atypical form of AD (the “visual variant of AD,” e.g., [21]), whereas others cite neuropathological evidence demonstrating that multiple pathologies can underlie the PCA syndrome (e.g., [16]). Inconsistency of terminology and usage likely reflects in part the interests or requirements of different investigators or research contexts. For example, syndromic classification is likely to be entirely appropriate for studies exploring behavioral interventions, whereas clinical trials of disease-specific pharmacological agents may additionally require consideration of the underlying molecular pathology. In the absence of criteria that clearly reflect this potential diversity of use, it remains unclear whether individuals with PCA should be included or excluded from conventional clinical trials for AD (e.g., owing to the potential unsuitability of the associated interventions, biomarkers, and/or outcome measures). Consequently, individuals affected by PCA risk being unable to access potentially helpful interventions owing to a lack of evidence regarding their effectiveness. Conversely, if criteria require evidence for AD pathology, individuals with PCA due to other causes may not be considered for behavioral interventional trials from which they may benefit. Finally, existing criteria provide an inadequate foundation from which to proceed with future studies exploring the factors influencing phenotypic heterogeneity and disease progression, acquiring evidence linking clinical phenotype to underlying pathology.

## 2. Aims

The present study describes the formation and deliberations of a PCA working party that aimed to establish a consensus opinion regarding the PCA syndrome. The goal of the work was to review, revise, and complement existing single-center diagnostic criteria to represent multidisciplinary and multicenter experience and knowledge. In light of the problems outlined previously and the challenges facing the PCA research field, a multilevel PCA classification framework is proposed for use in a number of different research contexts.

## 3. Methods

**Fig. 2:**
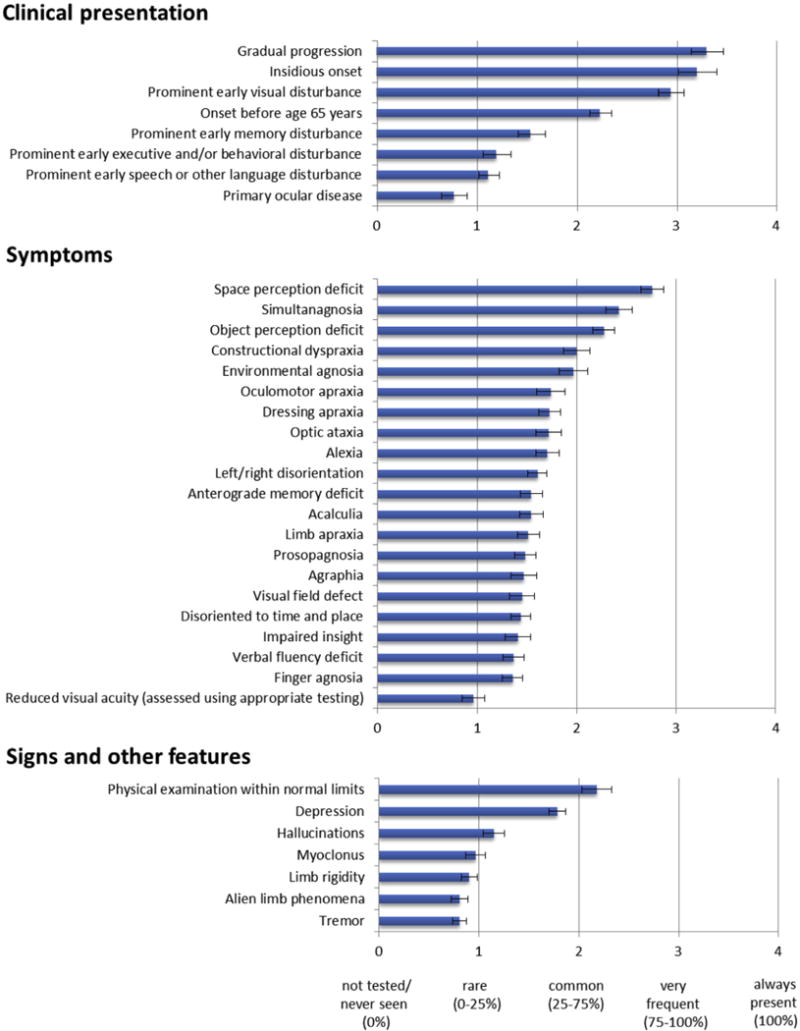
Mean and standard error ratings of clinical presentation features, symptoms, and signs in PCA, as rated by experts in the online survey. Abbreviation: PCA, posterior cortical atrophy.

Following a detailed review of the literature (S.J.C., M.L., J.M.S., G.D.R., M.N.R., and N.C.F. [22]), a PCA Working Party of experienced clinicians and researchers formed to develop a consensus statement regarding research criteria for PCA. Representatives of the group met at the Alzheimer’s Association International Conferences in Vancouver (July 2012; see [23]) and Boston (July 2013). An *Atypical Alzheimer’s Disease and Associated Disorders* Professional Interest Area (PIA) was subsequently constituted subsequently under the auspices of the International Society to Advance Alzheimer’s Research and Treatment. In June 2014, an online survey of Working Party and PIA members was conducted. Participants were requested to estimate the frequency of symptoms, signs, and features (never seen [0%], rare [0%–25%], common [25%–75%], very frequent [75%–100%], always present [100%]). Participants were also asked to rate their level of agreement with a series of statements regarding the conceptualization of PCA (Likert scale: 1 = strongly disagree, through to 7 = strongly agree). The survey was completed by 36 experienced group members with backgrounds in neurology, psychology, pathology, psychiatry, gerontology, and neuroscience (years since qualification: mode and median: 20–30 years, range: 1–>30 years; number of individuals with PCA encountered: median: 20–30, mode: 30–50, range: 1–>50). The results of the survey (see Fig. 2) and their implications for a consensus statement were discussed at the next PIA meeting (AAIC, Copenhagen, July 2014). The consensus statement was subsequently drafted (S.J.C.) and developed with a small group of experts (N.C.F., J.M.S., G.D.R., W.M.v.d.F., M.M., B.C.D., R.V., and J.S.S.), and a revised version circulated to the PCA Working Party and selected PIA members for their detailed feedback before final discussion and agreement (AAIC, Washington, July 2015). The final version was approved by all authors.

## 4. Classification framework

**Fig. 3:**
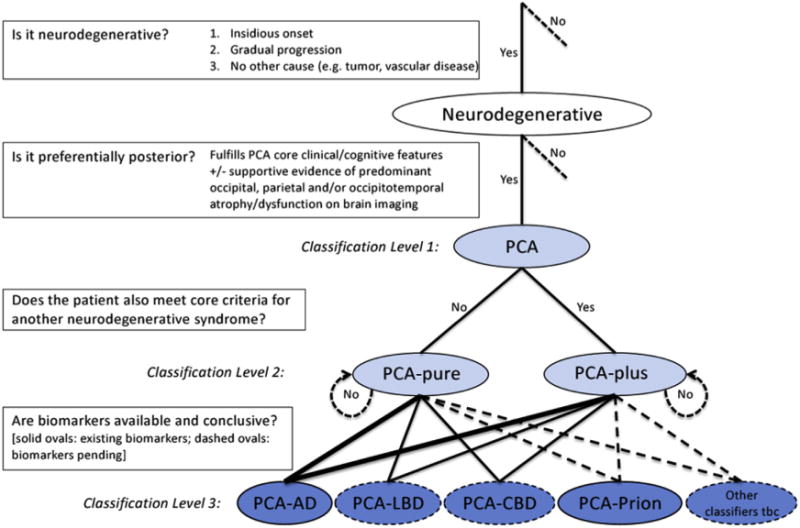
Diagnostic process and PCA classification. Key diagnostic questions at each level are shown in boxes. Syndrome-level descriptions (classification levels 1 and 2) are lightly shaded and disease-level descriptions (classification level 3) are darkly shaded. Among the disease-level classifications, PCA-AD and PCA-prion (solid ovals) are distinguished from PCA-LBD and PCA-CBD (dashed ovals) owing to the current availability of in vivo pathophysiological biomarkers. Other disease-level classifications may be appropriate (e.g., a patient with PCA plus visual hallucinations may have LBD-variant of AD) or anticipated (e.g., PCA attributable to GRN mutations). The thickness of lines connecting classification levels 2 and 3 is intended to reflect the status of AD as the most common cause of PCA. Abbreviations: AD, Alzheimer’s disease; CBD, corticobasal degeneration; LBD, Lewy body disease; PCA, posterior cortical atrophy; tbc, to be confirmed.

A three-level classification framework for PCA is described in Fig. 3. Level 1 establishes that the presenting problem has a neurodegenerative basis and a posterior cortical focus, based on the identification of the core clinical and cognitive features that define the PCA syndrome, plus supportive neuroimaging evidence if available. Further core features include evidence of insidious onset and gradual progression. Exclusion criteria include evidence of a brain tumor or other mass lesion, significant vascular disease including focal stroke, primary ocular disease, or other identifiable causes for cognitive impairment, but only where independently sufficient to explain the clinical and cognitive syndrome. Level 2 establishes whether the presentation is one of pure PCA or whether the patient meets the core criteria for both the PCA syndrome and an additional neurodegenerative syndrome (in the absence of biomarkers). Level 3 involves a more formal determination of the underlying cause of the PCA syndrome, based on pathophysiological biomarker evidence. Levels 1 and 2 yield a syndrome-level description of the presenting complaint. Level 3 yields a disease-level description. Levels 1–3 are outlined in greater detail in the following.

### 4.1. Classification level 1: The core features of the PCA syndrome

As defined in the perspective article which followed the first consensus meeting, “PCA is a clinico-radiological syndrome characterized by progressive decline in visual processing and other posterior cognitive functions, relatively intact memory and language in the early stages, and atrophy of posterior brain regions” ([24], p. 463). The core clinical, cognitive, and (optional supportive) neuroimaging features and exclusion criteria for PCA are listed in Table 1. These early or presenting features are listed in order of (descending) frequency at first assessment in line with the quantitative ratings provided by online survey participants (see Fig. 2). The list of cognitive features is a summation of all features listed in Mendez et al. [14] and Tang-Wai et al. [15].

The clinical and exclusion criteria constrain the definition of PCA to individuals with a neurodegenerative condition. The semi-arbitrary stipulation of three or more cognitive features is designed to ascertain evidence of a cluster of posterior cognitive deficits and reduce misclassifications based on overinterpretation of a single complaint or abnormal test score. The stringency of this stipulation is also mitigated by the extensive list of potential features which fall broadly into the domains of basic visual, visuoperceptual, visuospatial, literacy, numeracy, praxis, and higher sensory functions. Many of these posterior cognitive deficits may have a pronounced impact on activities of daily living.

A critical element of the PCA cognitive profile is the contrast between the posterior cortical dysfunction and the relative sparing of other cognitive domains. This is aimed at distinguishing PCA from typical (amnestic) AD (episodic memory), logopenic-variant primary progressive aphasia (lvPPA; language), frontotemporal dementia and the AD phenotype variously labeled frontal variant AD, behavioral variant AD, or dysexecutive AD (which primarily manifests as impairments of executive functions, behavior, and personality). The concept of “relative sparing” is intentionally flexible to accommodate different assessment settings and tools. Operationalizing these criteria with recommended sets of brief and detailed cognitive tasks is a future objective of the working group, but the main principle is to reduce the impact of core deficits on assessment of these functions. For example, accurate testing of anterograde memory in people with PCA requires tests that avoid not only explicit visual demands (e.g., Rey–Osterrieth figure copy) but also more implicit visual demands on visually mediated processes such as mental imagery (e.g., verbal paired associate learning).

The neuroimaging features of PCA are intentionally broad to reflect the loose anatomical description of “posterior cortical atrophy,” with the working group regarding evidence of focal structural (e.g., atrophy on magnetic resonance imaging) or functional (e.g., hypometabolism on ^18^F-labeled fluorodeoxyglucose positron emission tomography or single-photon emission computed tomography) abnormality in the occipital, parietal, and/or occipito-temporo-parietal cortices as supportive of the clinico-radiological syndrome. The inclusion of neuroimaging evidence of posterior cortical atrophy or dysfunction as an optional, supportive feature rather than obligatory component of the syndrome-level description is consistent with previous criteria. This issue generated considerable debate, but maintaining the optional status was justified on both clinical (e.g., variable extent of atrophy at presentation) and practical grounds (e.g., accessibility of neuroimaging facilities; not wishing to exclude patients unable to undergo M.R. investigation from all PCA-related research). Where for practical reasons neuroimaging evidence cannot be obtained, research studies should specify the evidence used to support the classification of PCA. Another issue is that individuals with the visual variant of Creutzfeldt–Jakob disease typically decline rapidly such that obvious focal atrophy is not easily demonstrated [25–27]. We debated the utility of classifying such individuals within the PCA framework, which may only be appropriate for prion disease patients with an insidious (rather than rapid) progression ([16], subject [21,28]). It should also be noted that evidence provided by more recently established molecular imaging techniques is incorporated together with other in vivo biomarkers in the disease-level description (see classification level 3).

**Fig. 4:**
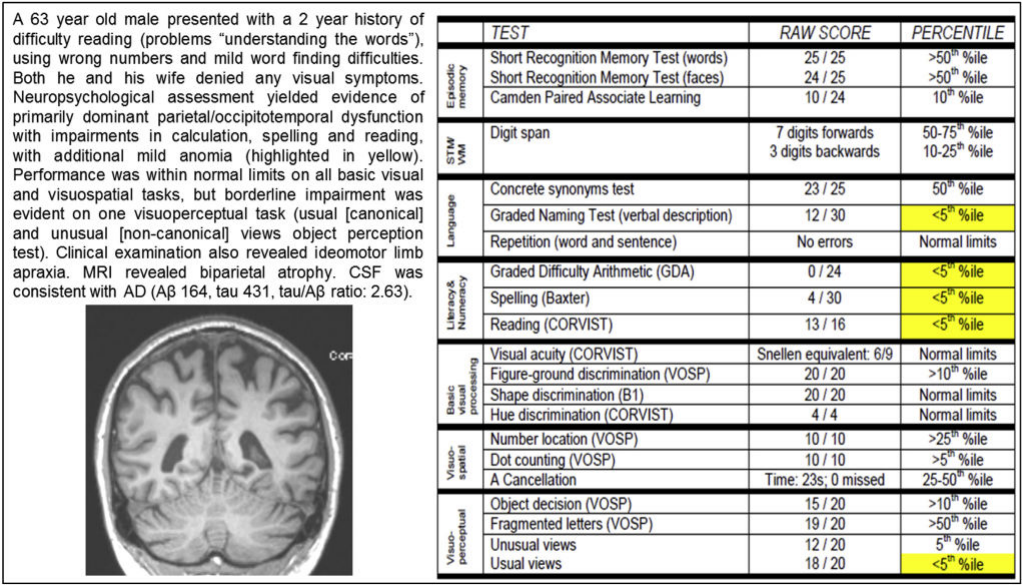
Case study of a PCA patient presenting with “nonvisual” symptoms. Abbreviation: PCA, posterior cortical atrophy.

The clinical, cognitive, and exclusion criteria (discussion of neuroimaging criteria mentioned previously) are largely consistent with existing single-center definitions of the syndrome [14,15]. Working group discussions elicited broad agreement regarding the specific features that constitute PCA and there was a strong reluctance to radically alter preceding descriptions of the syndrome, which has become a well-established clinical concept. Nonetheless, one point of note is the primacy given to visual impairment in the earlier criteria (Mendez et al: “Presentation with visual complaints with intact primary visual functions”; Tang-Wai et al: “Presentation of visual complaints in the absence of significant primary ocular disease explaining the symptoms” [14,15]). In the proposed consensus statement, this criterion is broadened to “Prominent early disturbance of visual plus/minus other cognitive functions with a presumed posterior location.” This reflects the position of 65% of the online survey group who agreed (compared with 15% disagreeing and 20% neither agreeing nor disagreeing) with the statement “Progressive focal disorders of nonvisual posterior cognitive functions (e.g., apraxia, agraphia, acalculia) can also be classified as PCA in some research contexts.” The working group rejected further broadening of the criterion to “Prominent early disturbance of visual and/or other posterior cognitive functions” on the basis that (1) removal of the visual criterion might lead to unhelpful diagnostic confusion between PCA and corticobasal syndrome (CBS), lvPPA and other syndromes, (2) very detailed neuropsychological testing of patients presenting with focal posterior nonvisual complaints typically uncover evidence of subtle impairments in visual cognition (see Fig. 4), and (3) some “nonvisual” complaints may be partly rooted in visual dysfunction (e.g., writing impairments partly attributable to disordered mental imagery for letters).

### 4.2. Classification level 2: Pure PCA and PCA with additional features (PCA-plus)

In classification level 2, a division is drawn between individuals who solely meet the criteria for PCA (PCA-pure) and individuals who exhibit additional features consistent with other recognized neurodegenerative syndromes (PCA-plus). All individuals must fulfill the criteria for the core clinico-radiological syndrome (level 1), with the PCA-pure/PCA-plus distinction made on the basis of nonfulfillment/fulfillment of additional core clinical criteria for lvPPA, CBS, or another neurodegenerative syndrome (see Table 2). The cited examples are based on recognized diagnostic criteria for the clinical syndromes of dementia with Lewy bodies [29] and CBS [30,31].

Classification level 2 exists as a buffer zone between a broad, purely symptomatic definition of PCA (level 1) and disease-level descriptions of the different clinico-biological entities (supported by biomarker evidence) which fall under that syndromic umbrella (level 3). This intermediate classification stage is motivated by a combination of in vivo biomarker and postmortem pathological data, clinical opinion, and research practicality. In vivo biomarker and postmortem pathological data from published case series indicate that the vast majority of reported cases of PCA are attributable to AD (see [15,16,32–34]). Reflecting such data, clinical opinion has tended toward regarding PCA primarily or even solely as an atypical phenotype of AD, many defining PCA as “the visual posterior variant of AD.” Accordingly, some working group members questioned whether features suggestive of non-AD pathologies, such as hallucinations and cognitive fluctuations suggestive of the histopathologically defined entity of Lewy body disease (LBD), should even be incorporated as exclusion criteria in the core definition of PCA. The PCA-pure/PCA-plus distinction permits clinicians or researchers to restrict study inclusion to a subpopulation who may be more likely to have AD as the underlying pathology, while at the same time acknowledging the lack of one-to-one correspondence between syndrome and pathology (e.g., patients fulfilling PCA and CBS criteria whose impairments are attributable to AD). The PCA-plus classification may also capture individuals who show additional features owing to mixed pathology or moderate to high subcortical vascular burden. Such debate may be moot in most situations where in vivo biomarkers are available. However, a full examination of the relationship between the PCA phenotype and underlying pathology will require documentation of the fulfillment/nonfulfillment of both core PCA and additional syndromic criteria. There are also methodological limitations concerning biomarkers reflected in variability of results across laboratories [35,36]. Besides, molecular imaging and cerebrospinal fluid analysis are not available for all individuals in all centers, and pathophysiological biomarkers are available for a limited number of diseases and it is only the diagnostic criteria for AD and FTD incorporate them. Ultimately, the Working Party aimed to produce consensus guidelines that have utility in every research setting. Thus, the concepts PCA-pure and PCA-plus are advanced as a simple, practicable method for improving the consistency of inclusion criteria in studies and refining PCA samples in situations where biomarker data are not available. Also, International Working Group criteria (IWG2) [20] do not provide a formal classification for individuals with a clinical presentation consistent with PCA in whom in vivo biomarkers are not available. The intermediate classification of PCA-pure/PCA-plus is aimed at facilitating the inclusion of participants in research studies even in the absence of direct evidence of underlying pathophysiological process. The practical implication of this formulation is that the concept of PCA-pure or -plus (level 2) may be largely redundant where biomarker evidence is available, where the expectation would be classification at disease-specific level 3 (see the following).

### 4.3. Classification level 3: Diseases causing PCA

Classification level 3 provides disease-level descriptions of PCA reflecting available evidence of the underlying pathology. Diagnostic criteria for PCA attributable to AD (PCA-AD), Lewy body disease (PCA-LBD), corticobasal degeneration (PCA-CBD), and prion disease (PCA-prion) are described in Table 3. The definition of PCA-AD is consistent with the IWG2 [20,37,38] definitions of AD, which require both the presence of an appropriate clinical phenotype and a pathophysiological biomarker consistent with the presence of AD pathology. However, pathophysiological biomarkers are only currently available for AD and prion disease (although not yet incorporated into formal diagnostic criteria for prion disease; see solid and dashed ovals in Fig. 3). Consequently, the disease-level descriptions provided in Table 3 are inequitable, with attribution of an in vivo diagnosis of PCA-LBD and PCA-CBD pending the development of suitable biomarkers. In these cases, use of the terms probable PCA-LBD and probable PCA-CBD may be appropriate where cases fulfilling both the relevant core clinical criteria are found to be negative for AD biomarkers. As noted in Fig. 3, other disease-level classifications may also be appropriate for individuals with mixed or multiple pathologies (e.g., a patient with PCA plus visual hallucinations could have LBD-variant of AD and therefore be more appropriately labeled PCA-AD/LBD; co-occurrence of AD and PSP: [39]) or required in future (e.g., PCA attributable to GRN mutations; [40]). Similarly, additional markers may become available to support existing and new classifications. Future iterations of the PCA consensus statement must consider these issues and revise the classifications in accordance with biomarker development and emerging clinical reports.

The division between syndrome-level and disease-level descriptions was supported by 88% of PCA Working Party members surveyed who agreed with the statement “Research criteria should discriminate the clinical syndrome (PCA) and the associated disease (e.g., PCA-AD, PCA-LBD).” The discussion of the syndrome/disease issue at consensus meetings reflected the balance sought between developing a flexible labeling system allowing for a wide variety of different research applications (e.g., disease-specific trials vs. nonpharmacological interventions targeting cognition, behavior, or function) and avoiding confusion by permitting a PCA subgroup (e.g., those fulfilling criteria for PCA and CBS) to skew the description of PCA. It is also important to note that the four proposed disease-level descriptions do not have the same frequency. Published data suggest that AD is overwhelmingly the most common underlying cause of PCA (e.g., [15,16,32–34,41,42]). Thus, in the absence of features to suggest an alternative diagnosis, AD is a priori the most likely underlying cause. The thickness of lines connecting classification levels 2 and 3 in Fig. 3 are intended to reflect the status of AD as the most common cause of PCA. It is also noted that in all cases, pathological confirmation of the underlying pathology is regarded as the “gold standard” and assigned the prefix definite.

There are a number of research contexts in which it may be important to identify the most likely underlying pathology associated with the PCA syndrome. The proposed disease-level descriptions of PCA may be of use in disease-specific clinical trials (by providing rationale the inclusion of PCA subjects in AD trials), in descriptive epidemiological studies investigating genetic and other determinants of phenotypic heterogeneity in AD and non-AD dementias, and in disease progression studies.

## 5. Further specification of PCA in a variety of research contexts

The classification system described previously provides syndrome-level and disease-level definitions of PCA for use in a variety of research contexts. However, there are a number of past and future contexts in which additional consensus descriptors might have value. Two important scenarios are staging the syndrome severity and describing phenotypic heterogeneity within PCA. In the following, we discuss this need, alongside the current labels related to PCA and their usage. We stop short of proposing an extended PCA lexicon but explicate how future research might prompt or guide a formal proposal of terms. All of the following scenarios consider the putative case of individuals who did, do, or might fulfill the core PCA criteria described previously at some point past, present, or future, plus the additional requirements listed in the following.

## 6. PCA stages

**Fig. 5:**
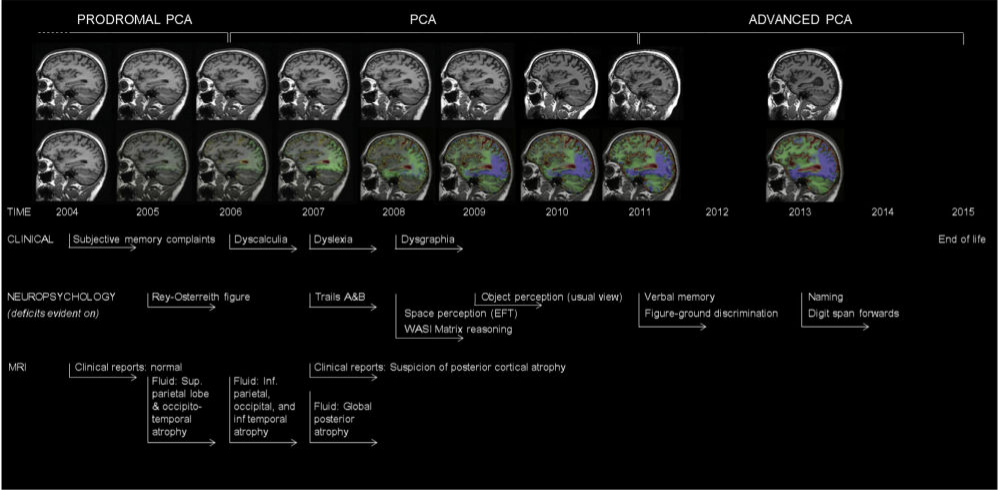
Longitudinal clinical, neuropsychological, and neuroimaging profile of an individual with pathologically proven PCA-AD showing example timelines for the provisional stages of prodromal PCA, PCA, and advanced PCA. Serial MR images (top row) show a sagittal view of the patient’s right hemisphere for all nine visits. Repeat scans were fluid-registered to the baseline image, and color-coded voxel-compression maps were produced (bottom row). The scale shows the percentage volume change per voxel (−20% to 20%) with green and blue representing contraction and yellow and red representing expansion. See Kennedy et al. [43] for a more detailed case description. Abbreviations: AD, Alzheimer’s disease; MR, magnetic resonance; PCA, posterior cortical atrophy.

One issue which may motivate an extension of PCA terminology concerns how researchers describe PCA at different stages of progression. To illustrate the issue, 11-year longitudinal data are presented on an individual who came to attention as a healthy research participant but subsequently developed PCA (see Fig. 5). The description of this patient at different points along the disease pathway is considered in the following alongside consideration of provisional terms.

- Prodromal/suspected/possible PCA: This term (see alternatives in the following) might be ascribed (in some circumstances, alongside other differential diagnoses) to individuals exhibiting subtle deficits in posterior cortical functions which are too mild or few (<3) to fulfill the core PCA criteria mentioned previously. As only individuals proceeding to a diagnosis of PCA could reliably be labeled prodromal PCA, this stage in the evolution of PCA would most likely be identified retrospectively in longitudinal studies (see Fig. 5). In other situations, alternative labels such as “suspected PCA” or “possible PCA” might be preferred. The concept of prodromal PCA is motivated by the assumption that a proportion of individuals with prodromal AD (IWG criteria; 37; clinical symptoms present but insufficient to affect instrumental activities of daily living) will be in the early clinical stages of PCA. By definition, the clinico-radiological syndrome PCA cannot be defined at the preclinical asymptomatic at-risk state for AD (IWG [38]) or stage 1 or 2 preclinical AD (NIA-AA [44]) where cognitive impairment is absent. It is also of note here that some individuals with PCA may not ever meet NIA-AA definitions of mild cognitive impairment (MCI), owing to the impact of even subtle posterior cortical dysfunction on everyday functional tasks. MCI criteria state “These cognitive changes should be sufficiently mild that there is no evidence of a significant impairment in social or occupational functioning” (p. 272), and “it must be recognized that atypical clinical presentations of AD may arise, such as the visual variant of AD (involving PCA) or the language variant (sometimes called logopenic aphasia), and these clinical profiles are also consistent with MCI due to AD” (p. 272). However, mild posterior cortical dysfunction can have a profound impact on certain everyday functions (e.g., driving). Although comparing levels of “severity” across different cognitive domains is difficult, it may be that the relative impact of mild cognitive deficits on everyday function may vary between typical and atypical AD phenotypes.
- PCA: The second stage of progression might simply be labeled PCA and could be entirely consistent with the definition of PCA provided previously in classification level I (namely fulfillment of the clinical, cognitive, neuroimaging, and exclusion criteria listed in Table 1). The only point of expansion is that, just as prodromal PCA would not necessarily equate to MCI, so PCA would not necessarily equate to dementia. Many patients with PCA for a time only show impairment in one of the five listed domains in McKhann et al. ([19]; i.e., evidence of visuospatial but not memory, reasoning, language, or personality/behavior deficits). At this stage, such cases may not fulfill the various rules for formal classification as dementia, and therefore diagnosis for which dementia is a prerequisite, namely probable AD dementia, possible AD dementia, or possible AD dementia with evidence of the AD pathophysiological process.
- Advanced PCA: This third provisional staging term could be used to describe individuals who have or would have previously met criteria for PCA but in whom disease progression has led to impairments in other aspects of cognitive function (i.e., episodic memory, language, executive functions, behavior, and personality). Advanced PCA might most typically be observed in individuals in whom either (1) visual ± nonvisual posterior dysfunction with relative preservation of these other cognitive skills was the primary complaint, but memory, language, executive, and/or behavior/personality deficits have now progressed and are also significantly impaired or (2) impairments in visual ± nonvisual posterior functions and one or more of these other cognitive skills were evident at presentation but the clinical history and/or other evidence indicate that posterior cortical deficits were the primary complaint (i.e., the patient did not present/was not assessed at the earlier stage when PCA could have been diagnosed). The term advanced PCA might be applicable to a number of PCA patients described in the existing literature. For example, in a study of PCA basic visual function [45], all 21 patients fulfilled Mendez et al. [14] and Tang-Wai et al. [15] criteria and had current or previous evidence on formal neuropsychological assessment of impaired visual function with relatively preserved (normal range) scores on at least one test of episodic memory. However, at the time of the experimental study, 5/21 (24%) had progressed to a point where episodic memory test scores fell below the normal range, with 12/21 (57%) showing deficits on naming from description (executive functions, behavior, and personality were not assessed formally). The advanced PCA concept is particularly relevant to the characterization of research participants, prognostic and longitudinal studies, clinical management and care planning, and for educating to patients and their caregivers.

## 7. Heterogeneity within the PCA spectrum

Another issue that may motivate an extension of PCA terminology is the challenge of describing the considerable heterogeneity that exists within the PCA spectrum. A number of subtypes have been described previously based on the distinct presentation of individuals or small series of patients (e.g., [46,47]). However, other studies have suggested that heterogeneity in PCA is best conceptualized as a multidimensional phenotypic space rather than as a series of distinct subtypes (see [17,45,48–50]). The Working Party concluded that there is currently insufficient cognitive or neuroimaging evidence to support the existence of discrete PCA subtypes and would recommend research to determine whether subtypes can be found in distinct patterns of cognitive impairment, atrophy, and disease progression. Nonetheless, the following provisional set of qualitative descriptions of different positions within this putative phenotypic space is provided to encourage debate concerning the consistent labeling among research studies and centers and to permit individual presentations to be described or quantified in terms of proximity to one or more of these prototypes.

- Biparietal (dorsal) variant: This subtype has been described as “a biparietal variant defined by the presence of early, predominant, and progressive difficulty with visuospatial function, features of Gerstmann syndrome, of Balint syndrome, limb apraxia, or neglect” [20]. This IWG2 designation is broadly consistent with other definitions of a biparietal atrophy syndrome (e.g., [29,46,47]).
- Occipitotemporal (ventral) variant: The biparietal variant of PCA has been contrasted with a more ventral occipitotemporal variant defined by the presence of an early, predominant, and progressive impairment of visuoperceptive functions or of visual identification of objects, symbols, words, or faces’ (IWG2: [20,29,46,47,51]). Such a category might include descriptions of patients with a progressive perceptual prosopagnosia (e.g., [52]).
- Primary visual (caudal) variant: Arguably even rarer than the biparietal or occipitotemporal syndromes is a more caudal primary visual syndrome characterized by primary visual failure, impairment of basic perceptual abilities, and bilateral occipital atrophy [29,47,53,54]. Owing to the early damage of primary visual cortex, as contrasted with the early involvement of association cortices seen in the biparietal and occipitotemporal variants, PCA patients with this primary visual variant are most distinct clinically and phenotypically from “typical AD.” The primary visual variant is rarely reported and not mentioned explicitly in IWG2. However, this lack of evidence may partly reflect less frequent testing/recording of basic visual functions compared with higher order object and space processing deficits (of which patients are also naturally more likely to complain). An analysis of basic visual functions (form detection, form discrimination, form coherence, motion coherence, color discrimination, single-point localization) found impaired performance in at least one basic visual skill in all 21 PCA patients tested, suggesting elementary visual deficits are a more common cause of or contributor to higher order object and space processing problems than is typically recognized [45].
- Dominant parietal variant: The presentation of visual complaints is a core feature of both the proposed consensus and existing single-center diagnostic criteria. However, not all PCA patients refer explicitly to visual problems among their primary complaints and may present with predominant impairment of other posterior cortical functions, such as calculation, spelling, and praxis [7,30,55–57]. Such presentations are most commonly associated with biparietal atrophy with relatively greater involvement of the dominant/left posterior cortices (see Fig. 4). In our survey, 67% of PCA Working Party members considered such individuals to fall within the PCA spectrum. This position, and the duality of the current criteria necessitating both “Prominent early disturbance of visual ± other posterior cortical functions” and presence of a cluster of three or more cognitive deficits which could appear “nonvisual” (e.g., acalculia, agraphia, and apraxia), might at first glance appear inconsistent. However, it is important to bear in mind that there is not a one-to-one correspondence between cognitive tests and the underlying cognitive processes. Many apparently “nonvisual” tasks (e.g., calculation) do make demands on visual imagery and spatial processing skills. Furthermore, as noted previously, very detailed neuropsychological testing of patients presenting with focal posterior nonvisual complaints typically uncovers evidence of subtle impairments in visual cognition (see Fig. 4). Finally, “nonvisual” presentations are one reason why the proposed criteria stipulate the need for a constellation of deficits (three or more cognitive features), to exclude for example cases of selective apraxia which could have a more anterior basis.

Naturally, there are multiple ways of classifying clinical heterogeneity within the PCA spectrum. An alternative way to discriminate putative subphenotypes would be to refer exclusively to the organization of the visual system (e.g., ventral and dorsal streams). Certain presentations may also merit the combination of different types of descriptive terminology (e.g., prodromal occipito-temporal variant PCA). It is anticipated that descriptions of PCA variants may be relevant to brain-behavior studies, phenotype characterization work (e.g., clarifying the degree of homogeneity/heterogeneity within the PCA spectrum), and examinations of phenotype progression (e.g., establishing the order of loss of different aspects of cortical visual processing). Descriptions of PCA variants may also be useful in the design and use of nonpharmacological interventions, aids, and strategies which are geared toward helping individuals with PCA cope with or ameliorate problems associated with a specific aspect of cognitive function (e.g., cues which aid object localization but not object identification). However, it should be stressed again that these descriptions are preliminary characterizations of positions within a spectrum of continuous variation.

## 8. Conclusions

We have proposed a PCA classification framework with both syndrome- and disease-level descriptions for use in a number of different research contexts. The strong agreement over the features which constitute the core clinico-radiological syndrome (classification level 1) re-affirmed the Working Party’s original aim of offering a consensus statement refining and advocating specific uses and adaptations of the term PCA rather than any wholesale alteration or redescription of the syndrome. Accordingly, the working party regard classification levels 2 (PCA-pure/PCA-plus) and 3 (PCA-AD, PCA-LBD, PCA-CBD, PCA-Prion) as extensions to rather than replacements for the previous single-center diagnostic criteria, aimed at increasing the utility and specification of the PCA concept in a variety of research settings.

The three-level classification system provides a standard against which clinicians, academics, reviewers, and editors may evaluate and compare study populations and inclusion/exclusion criteria across previous publications and future studies. Although the proposed core features permit of heterogeneity within the syndrome consistent with the amassed clinical experience, reducing the variability between sites and studies caused by inconsistencies of terminology and diagnostic criteria will benefit a variety of future studies. Refining study populations and minimizing clinico-pathological “noise” through both tighter diagnostic criteria and in vivo biomarkers is particularly important in the context of a relatively rare disorder such as PCA in which sample sizes are often limited.

A number of challenges remain for the PCA research field. A primary challenge is to understand sources and drivers of phenotypic heterogeneity among individuals with a common underlying pathology. For example, members of the atypical AD PIA have contributed to the largest analysis to date of genetic risk factors for PCA, yielding evidence of both an altered risk profile across known AD risk factors and possible novel loci some of which are associated with visual system development [58]. The current proposed consensus criteria for PCA may complement equivalent consensus diagnostic criteria for typical amnestic AD [19,20] and other atypical AD syndromes (e.g., lvPPA; [59]) to improve the robustness and replicability of future heterogeneity studies which may shed light on fundamental mechanisms of disease progression and propagation.

Second, although the relationships between syndrome and pathology are indubitably less complex than among, for example, the frontotemporal dementias, the boundaries between PCA, and related syndromes (e.g., CBS) require further clarification through quantitative investigation. For example, motor impairment (defined by asymmetrical left upper limb rigidity) has been found in 30% of a series of 44 patients all meeting the existing clinical criteria for PCA [60]. Visuospatial and visuoperceptual dysfunction in CBS patients has also been shown to predict AD pathology [30,61,62].

Third, a further practical objective is to establish a common framework for cognitive screening, neuropsychological examination, and selection of cognitive outcome measures for trials involving individuals with PCA. There is a particular need for clarity regarding the evaluation of episodic memory in PCA [63]. As noted previously, memory tests with explicit visual demands in encoding and/or retrieval (e.g., Rey–Osterrieth figure copy) are unsuitable. Less obvious are the more implicit visual demands of tests such as verbal paired associate learning that often draw on mental imagery. Two alternate forced choice recognition memory tests for words [64] are suitable for evaluating aspects of episodic memory in PCA. Evaluating alternative metrics would help optimize techniques for establishing and quantifying this critical distinction between PCA and typical amnestic AD, namely “prominent early disturbance of vision with relatively preserved anterograde memory.”

The proposed classification framework will not resolve these issues directly but may improve our ability to interpret findings across studies, increase the quality of clinical trials for AD, and provide a foundation for future collaborative work. There is a need to validate the reliability, sensitivity, and specificity of the proposed criteria, particularly to establish the quantitative relationships between the different levels of classification. The classification system will also likely require updating and revision, particularly based on emergence of new biomarkers and clinical evidence of PCA attributable to non-AD and mixed pathologies.

## Figures and Tables

**Table 1:** Core features of the PCA clinico-radiological syndrome (classification level 1)

Clinical, cognitive, and neuroimaging features are rank ordered in terms of (decreasing) frequency at first assessment as rated by online survey participants (see Fig. 2)
Clinical features:
Insidious onset
Gradual progression
Prominent early disturbance of visual ± other posterior cognitive functions
Cognitive features:
At least three of the following must be present as early or presenting features ± evidence of their impact on activities of daily living:
Space perception deficit
Simultanagnosia
Object perception deficit
Constructional dyspraxia
Environmental agnosia
Oculomotor apraxia
Dressing apraxia
Optic ataxia
Alexia
Left/right disorientation
Acalculia
Limb apraxia (not limb-kinetic)
Apperceptive prosopagnosia
Agraphia
Homonymous visual field defect
Finger agnosia
All of the following must be evident:
Relatively spared anterograde memory function
Relatively spared speech and nonvisual language functions
Relatively spared executive functions
Relatively spared behavior and personality
Neuroimaging:
Predominant occipito-parietal or occipito-temporal atrophy/hypometabolism/hypoperfusion on MRI/FDG-PET/SPECT
Exclusion criteria:
Evidence of a brain tumor or other mass lesion sufficient to explain the symptoms
Evidence of significant vascular disease including focal stroke sufficient to explain the symptoms
Evidence of afferent visual cause (e.g., optic nerve, chiasm, or tract)
Evidence of other identifiable causes for cognitive impairment (e.g., renal failure)

Abbreviations: PCA, posterior cortical atrophy; MRI, magnetic resonance imaging; FDG-PET, ^18^F-labeled fluorodeoxyglucose positron emission tomography; SPECT, single-photon emission computed tomography.

**Table 2:** Classification of PCA-pure and PCA-plus (classification level 2)

PCA-pure
Individuals must fulfill the criteria for the core clinico-radiological PCA syndrome (level 1), and not fulfill core clinical criteria for any other neurodegenerative syndrome.
PCA-plus
Individuals must fulfill the criteria for the core clinico-radiological PCA syndrome (level 1) and also fulfill core clinical criteria for at least one other neurodegenerative syndrome, such as
Dementia with Lewy bodies (DLB)
Following the diagnostic criteria proposed by the DLB consortium (McKeith et al., 2005), individuals must exhibit two or more core features of DLBs (list A) or one or more core features (list A) and one or more suggestive features (list B):
A. Core features
• Fluctuating cognition with pronounced variations in attention and alertness
• Recurrent visual hallucinations that are typically well formed and detailed
• Spontaneous features of parkinsonism
B. Suggestive features
• Rapid eye movement (REM) sleep behavior disorder
• Severe neuroleptic sensitivity
• Low dopamine transporter uptake in basal ganglia demonstrated by SPECT or PET imaging
Corticobasal syndrome (CBS)
Following the modified CBS criteria proposed by Armstrong et al. (2013), a diagnosis of probable CBS requires asymmetric presentation of 2 of
a) limb rigidity or akinesia
b) limb dystonia
c) limb myoclonus
plus 2 of:
d) orobuccal or limb apraxia
e) cortical sensory deficit
f) alien limb phenomena (more than simple levitation)
Possible corticobasal syndrome may be symmetric and requires presentation of 1 of a–c plus 1 of d–f.

Abbreviations: PCA, posterior cortical atrophy; SPECT, single-photon emission computed tomography; PET, positron emission tomography.

**Table 3:** Diagnostic criteria for disease-level descriptions (classification level 3)

PCA-AD
Following IWG2 (Dubois et al., 2014), the classification of PCA-AD (and, by extension, of IWG2’s broader category of “atypical AD”) requires fulfillment of the PCA syndrome (classification level 1) plus in vivo evidence of Alzheimer’s pathology (at least one of the following):
• Decreased Aβ_1–42_ together with increased T-tau and/or P-tau in CSF
• Increased tracer retention on amyloid PET
• Alzheimer’s disease autosomal-dominant mutation present (in *PSEN1*, *PSEN2*, or *APP*)
If autopsy confirmation of AD is available, the term definite PCA-AD would be appropriate.
PCA-LBD
Molecular biomarkers for LBD are currently unavailable; therefore, an in vivo diagnosis of PCA-LBD cannot be assigned at present. For individuals who are both classified as PCA-mixed by virtue of fulfilling DLB clinical criteria and shown to be AD-biomarker negative, the term probable PCA-LBD may be appropriate. If autopsy confirmation of LBD is available, the term definite PCA-LBD would be appropriate. Other disease-level classifications may also be appropriate for individuals with mixed or multiple pathologies (e.g., PCA-AD/LBD).
PCA-CBD
Molecular biomarkers for CBD are currently unavailable; therefore, an in vivo diagnosis of PCA-CBD cannot be assigned at present. For individuals who are both classified as PCA-mixed by virtue of fulfilling CBS criteria and shown to be AD-biomarker negative, the term probable PCA-CBD may be appropriate. If autopsy confirmation of CBD is available, the term definite PCA-CBD would be appropriate.
PCA-prion
There are a number of promising biomarkers for prion disease (e.g., Orru et al., 2014; Jackson et al., 2014; McGuire et al., 2012), but these have yet to incorporated into diagnostic criteria. Pending this process, an in vivo diagnosis of PCA-prion may be feasible. If autopsy confirmation of prion disease is available or a known genetic form of prion disease has been determined, the term definite PCA-prion would be appropriate.

Abbreviations: PCA, posterior cortical atrophy; AD, Alzheimer’s disease; IWG2, International Working Group; CSF, cerebrospinal fluid; LBD, Lewy body disease; DLB, dementia with Lewy bodies; CBD, corticobasal degeneration; CBS, corticobasal syndrome.

## RESEARCH IN CONTEXT

1. Systematic review: Consensus statements about posterior cortical atrophy (PCA) were developed through a detailed literature review, the formation of an international multidisciplinary working party which convened on four occasions and a Web-based quantitative survey regarding symptom frequency and the conceptualization of PCA.
2. Interpretation: The proposed classification framework for PCA—the first consensus-based, multicenter statement—is proposed to (1) improve the uniformity of definition of the syndrome in a variety of research settings, (2) encompass both syndrome- and disease-level descriptions, (3) incorporate the recent development of AD pathophysiological biomarkers, (4) describe individuals with the PCA syndrome who are negative for AD pathophysiological biomarkers, and (5) define stages of syndrome severity and characterize phenotypic heterogeneity within the PCA spectrum.
3. Future directions: The framework and terminology may facilitate the interpretation of research data across studies, be applicable across a broad range of research scenarios (e.g., behavioral interventions, pharmacological trials), and provide a foundation for future collaborative work.
